# *Lactobacillus sakei* ADM14 Induces Anti-Obesity Effects and Changes in Gut Microbiome in High-Fat Diet-Induced Obese Mice

**DOI:** 10.3390/nu12123703

**Published:** 2020-11-30

**Authors:** Sung-Min Won, Siyu Chen, Seo Yeon Lee, Kyung Eun Lee, Kye Won Park, Jung-Hoon Yoon

**Affiliations:** Department of Food Science and Biotechnology, Sungkyunkwan University, 2066 Seobu-ro, Jangan-gu, Suwon 16419, Korea; lionbanana@skku.edu (S.-M.W.); csylovejsg@naver.com (S.C.); lhmseo95@naver.com (S.Y.L.); sally9237@naver.com (K.E.L.); kwpark@skku.edu (K.W.P.)

**Keywords:** *Lactobacillus sakei*, probiotics, anti-obesity, gut microbiome

## Abstract

The aim of our study was to evaluate the anti-obesity effects of *Lactobacillus sakei* (*L. sakei*) ADM14 administration in a high-fat diet-induced obese mouse model and the resulting changes in the intestinal microbiota. Prior to in vivo testing, *L. sakei* ADM14 was shown to inhibit adipogenesis through in vitro test and genetic analysis. Subsequently, mice were orally administered 0.85% saline supplemented or not with *L. sakei* ADM14 to high-fat diet group and normal diet group daily. The results showed that administration of *L. sakei* ADM14 reduced weight gain, epididymal fat expansion, and total blood cholesterol and glucose levels, and significantly decreased expression of lipid-related genes in the epididymal fat pad. Administration of *L. sakei* ADM14 showed improvement in terms of energy harvesting while restoring the Firmicutes to Bacteroidetes ratio and also increased the relative abundance of specific microbial taxa such as *Bacteroides faecichinchillae* and *Alistipes*, which are abundant in non-obese people. *L. sakei* ADM14 affected the modulation of gut microbiota, altered the strain profile of short-chain fatty acid production in the cecum and enhanced the stimulation of butyrate production. Overall, *L. sakei* ADM14 showed potential as a therapeutic probiotic supplement for metabolic disorders, confirming the positive changes of in vivo indicators and controlling gut microbiota in a high-fat diet-induced obese mouse model.

## 1. Introduction

Obesity, which is widespread worldwide, is a serious threat to public health, as a factor associated with many metabolic diseases [1]. Obesity is a complex disorder caused by interactions among genetic, environmental, and psychosocial factors [2]. In particular, the high-carbohydrate, high-fat modern diet is considered the leading cause of human obesity and metabolic syndrome [3]. Recent studies have shown that obesity is associated strongly with the bacterial gut microbiome, which is manifested in specific bacterial compositions and functional alterations [4]. These changes can also lead to dysbiosis, which occurs in obesity. Previous studies have shown that weight gain and metabolic disorders occur when gut microbiota of obese mice are inoculated into normal or germ-free mice [5]. Specific gut microbiota are associated with obesity, and the ratio of representative dominant strains Firmicutes and Bacteroidetes is a main factor. Changes in gut microbial community due to obesity are related to the ability of the microbes to perform dietary energy harvest [6] and to alter fatty acid metabolism in the adipose tissue and liver [7]. Changes in dominant strains cause differences in terms of energy harvesting, which can be one of the causes of metabolic problems. Therefore, the modulation of gut microbiota composition represents an attractive potential treatment for metabolic disorders [8].

Probiotics are living bacteria that maintain good health in the gastrointestinal tract and provide positive health effects to the host when consumed in proper amounts [9]. These beneficial effects include reduction of blood cholesterol and hypertension, modulation of the immune system, and management of intestinal inflammatory diseases. Several studies have shown that certain probiotics have biological effects on both human and animal adipocytes [8]. Probiotics have potential as an alternative supplement to treat metabolic diseases, and their manner of action is through changing the composition and function of the gut microbiota [10]. Inhibiting the synthesis of fatty acids and reducing inflammation in vivo through intervention of specific probiotics clearly demonstrate the function of probiotics [11]. Despite the beneficial effects of improving the intestinal environment and resolving gut microbiota dysbiosis, an overall understanding of probiotics is lacking.

Kimchi is a traditional Korean food made of various vegetables and seasonings. It contains various vitamins and minerals, and natural lactic acid bacteria derived from various vegetables and major ingredients are involved in fermentation. The fermentation of kimchi proceeds with various lactic acid bacteria, among which *Lactobacillus sakei* (*L. sakei*) is important and is the representative dominant bacteria [12]. In addition to inhibiting the growth of harmful bacteria, such as those associated with food poisoning [13], previous studies have shown that *L. sakei* modulates allergic Th2 responses and has immune regulation effects by increasing anti-inflammatory cytokine production [14,15]. In addition to immune control, *L. sakei* has been reported to have anti-obesity improvement effects through the inhibition of harmful bacteria [16].

In our previous study, the *L. sakei* ADM14 strain was isolated from kimchi and shown to have an anti-adipogenic effect, along with basic properties of probiotic strains such as viability in acid and bile salts and good adhesion to Caco-2 cells [17]. In this study, we confirmed the adipogenesis inhibition effect of *L. sakei* ADM14 in detail through in vitro test and confirmed the anti-obesity effect through various biomarkers in in vivo experiments. Additionally, the improvement effect of *L. sakei* ADM14 in obesity-induced dysbiosis was investigated. Based on the results, it was confirmed that *L. sakei* ADM14 is a potential therapeutic probiotic agent through the inhibition of adipogenesis and modulation of gut microbial community.

## 2. Materials and Methods

### 2.1. Bacterial Strain Preparation and Growth Conditions

*L. sakei* ADM14 was isolated from Andong sik-hae kimchi, one of the kimchi collected in Korea regions, and the isolation was conducted as described in a previously reported method [17]. Strain was cultured using de Man–Rogosa–Sharpe (MRS, BD Difco, Sparks, MD, USA) agar at 30 °C for 24 h. The strain was stored at −80 °C in 20% glycerol (Georgiachem, GA, USA) (*v*/*v*) as a cryoprotective agent until it was used in experiments.

To prepare lactic acid bacteria (LAB) extract for in vitro studies, extraction was conducted as described in a previously reported method [18]. Cells of the strain ADM14 were suspended in distilled water at a concentration of 100 mg/mL and sonicated 50 times for 5 s at a 10 s interval on ice using an ultrasonicator at an intensity of 35% (Sonics and Materials Inc., Newtown, CT, USA). The LAB extract was centrifuged at 13,000 rpm for 15 min at 4 °C. The supernatant was filtered through a 0.45 μm syringe filter (Sartorius Stedim Biotech GmbH, Göttingen, Germany) and lyophilized. The resulting powder was dissolved in distilled water to the desired experimental concentration.

To prepare the strain for oral administration in animal experiments, *L. sakei* ADM14 was cultured overnight at 30 °C in MRS broth. After culturing, it was inoculated (2%) into 3 L MRS broth. After incubation at 30 °C for 48 h, cells were harvested by centrifugation at 7000 rpm for 15 min and washed with sterile saline (0.85% NaCl, *w*/*v*). The harvested cells were lyophilized and suspended in sterile saline (0.85% NaCl, *w*/*v*) at a concentration of approximately 1 × 10^9^ CFU/0.2 mL for oral administration to mice.

### 2.2. Cell Culture and Adipocyte Differentiation Test

Pre-adipocyte 3T3-L1 cells used in the experiments were purchased from the American Type Culture Collection (Manassas, VA, USA). 3T3-L1 cells were maintained and differentiated into adipocytes as described in a previously reported method [19]. Sub-culture of 3T3-L1 cells was performed with Dulbecco’s modified Eagle’s medium (DMEM) containing 10% fetal calf serum (Hyclone, Logan, UT, USA) and antibiotics (penicillin and streptomycin solution, Hyclone) at 37 °C in a 5% CO_2_ atmosphere. For differentiation into adipocytes, confluent 3T3-L1 cells were cultured for 2 days at 37 °C in a 5% CO_2_ atmosphere in DMEM containing 10% fetal bovine serum (FBS, Hyclone), antibiotics (penicillin and streptomycin solution, Hyclone), 0.5 mM 3-isobutyl-1-methylxanthine (Sigma-Aldrich, St. Louis, MO, USA), 1 M dexamethasone (Sigma-Aldrich), and 10 μg/mL insulin (Sigma-Aldrich). Every 2 days, the medium was replaced with fresh DMEM containing FBS and insulin, and LAB cell extract suspended in sterile distilled water to each experimental concentration was treated during adipocyte differentiation. Sterile distilled water was treated as a control. After 6 days of differentiation, 3T3-L1 cells were fixed with 3.8% paraformaldehyde (Sigma-Aldrich) in phosphate-buffered saline (pH 7.2) at room temperature overnight and stained with Oil Red O (Sigma-Aldrich). To quantify intracellular lipid accumulation content, stained cells were resolved in isopropanol (Sigma-Aldrich) and measured with an Eon Microplate spectrophotometer (Biotek, Seoul, Republic of Korea) at 520 nm.

### 2.3. Cell Viability Test

Cytotoxicity and cell viability were confirmed by MTS assay (Promega, Madison, WI, USA) according to the manufacturer’s protocols. Pre-adipocyte 3T3-L1 cells were seeded at 1 × 10^4^ cells per well in 96-well culture plates. The LAB extract was treated and cultured at 37 °C in a 5% CO_2_ atmosphere for 24 h. After overnight incubation, 20 µL of CellTiter 96^®^ AQueous One Solution Reagent (Promega) was added into each well and incubated at 37 °C under 5% CO_2_ for 4 h. Subsequently, the absorbance of each well was measured with an Eon Microplate spectrophotometer (Biotek, Seoul, Korea) at 490 nm.

### 2.4. Animals, Diets, and Experimental Design

The care and study of the experimental mice followed protocols of the Animal Care and Use Committee of the College of Biotechnology at Sungkyunkwan University (approval date: 08-11-2017, approval number: SKKUIACUC-17-9-11-1). Male C57BL/6J mice (*n* = 24) aged 5 weeks were purchased from RaonBio Inc. (Yongin-si, Gyeonggi-do, Republic of Korea). All mice were cared for under controlled temperature and humidity (24 ± 2 °C, 50% ± 10%) with a 12 h light/dark cycle. After a 1 week acclimation period, the mice were randomly divided into 4 groups (*n* = 6/group). Groups fed normal diet (normal chow, 10% of energy from fat, 16.1 kJ, RaonBio Inc.) were named ND group and NDA group. The groups fed high-fat diet (HFD, 60% of energy from fat, 21.9 kJ, RaonBio Inc.) were named HD group and HDA group. Each diet was fed for 10 weeks. The NDA group and HDA group were groups in which *L. sakei* ADM14 was administered orally for 10 weeks at the same time as the diet. *L. sakei* ADM14 was administered by oral gavage daily at a concentration of 10^8^–10^9^ CFU per 200 μL of 0.85% saline. ND group and HD group also consumed 200 μL of 0.85% saline by oral gavage. Food intake and body weight were measured weekly for 10 weeks. The food efficiency ratio (FER) was expressed as total body weight gained from the diet divided by total diet consumed during the animal experiments. Total calorie intake was calculated as total amount of diet consumed during the animal experiment multiplied by caloric value of the diet. After 10 weeks, the experimental mice were fasted for 16 h and sacrificed under anesthesia. After sacrifice, the visceral organs (liver, spleen, kidney, cecum) and epididymal fat pad were collected and weighed. Epididymal fat pads were preserved by freezing in liquid nitrogen for quantitative real-time polymerase chain reaction analysis. Blood was collected via cardiac puncture and was centrifuged for 10 min at 3000 rpm for serum separation.

### 2.5. Analysis of Blood Biochemical Parameters

Alanine transaminase (ALT), aspartate transaminase (AST), serum total-cholesterol, and glucose concentrations were measured using a biochemical automatic analyzer (Hitachi-7180, Hitachi Medical, Japan) in accordance with the IFCC (International Federation of Clinical Chemistry) standard method.

### 2.6. RNA Extraction and Quantitative Real-Time Polymerase Chain Reaction (RT-PCR)

Total RNA was extracted from 3T3-L1 cells and mouse fat tissue using an RNeasy mini kit (Qiagen, Hilden, Germany) and TRIzol (Invitrogen, Carlsbad, CA, USA) according to the manufacturer’s protocol. First-strand complementary DNA was synthesized from 0.5 μg of extracted total RNA using ReverTra Ace Master Mix (TOYOBO, Osaka, Japan). The RT-PCR was performed in a mixture containing Power SYBR Premix ExTaq (RP041A; Takara, Shiga, Japan), primers, and cDNA using a Thermal Cycler Dice instrument (Takara). Gene expression was normalized by housekeeping gene 36B4. The sequences of primers used are described by Song et al. [19]. Primers are specified in Supplementary Table S1.

### 2.7. 16S rRNA Gene Sequence Analysis of Gut Microbiota and Bioinformatics

To analyze of the microbiota, total genomic DNA was extracted from cecal content using the QIAamp DNA stool mini kit (Qiagen) according to the manufacturer’s protocol. The first amplification was performed targeting the V3 to V4 regions of the 16S rRNA gene [20]. The second amplification was performed by attaching an Illumina NexTera barcode to the amplification product. Sequencing was performed with an Illumina MiSeq Sequencing system (Illumina, San Diego, CA, USA) according to the manufacturer’s methods (Chunlab, Inc., Seoul, Korea). Taxonomic profiling was performed using the MiSeq pipeline of the Illumina platforms method described by Chunlab, Inc. After pre-processing [21], which included a quality check and filtering of low-quality (<Q25) reads, paired-end sequence data were merged using PANDAseq [22]. Non-specific amplicons that do not encode 16S rRNA were detected using HMMER’s hmmsearch program [23], and non-redundant reads were extracted by UCLUST-clustering [24]. The EzBioCloud database was used for taxonomic assignment using USEARCH (8.1.1861_i86linux32) [24]. Chimeras were detected and assessed using the UCHIME program [25] and reference sequences of the EzBioCloud database. Sequence data were clustered using CD-HIT [26] and UCLUST, and operational taxonomic units (OTUs) were chosen.

Alpha diversity indices were calculated using the OTU information. Beta diversity indices were analyzed by generalized UniFrac principal coordinate analysis (PCoA) and the Unweighted Pair Group Method with Arithmetic Mean (UPGMA). The relative abundance (%) of bacteria at the phylum level, class level, and family level, as well as the core microbial communities of each group, were calculated and compared.

### 2.8. Quantitative Analysis of Short-Chain Fatty Acids (SCFAs)

SCFA measurements were performed according to the protocol published by Mendes et al. [27]. Cecal content was weighed at 50 mg into a 1.5 mL tube and homogenized in 100 µL of distilled water. Then, 40 mg of sodium chloride (Sigma-Aldrich), 20 mg of citric acid (Merck, Darmstadt, Germany), 40 µL of 1M hydrochloric acid (Georgiachem), and 200 µL of butanol (Merck) were added and vortexed for 5 min. The supernatant was separated by centrifuging the tube at 13,000 rpm for 15 min. For gas chromatography analysis, the supernatant was transferred to a microtube, and 5 µL was injected into the gas chromatograph. To quantify SCFAs, a calibration curve was constructed for a concentration range of 0.015–1 mg/mL. Chromatographic analyses were performed using an Agilent 6890 system with ExChrom software equipped with a 7683B automatic liquid sampler (Agilent Technologies Inc., Philadelphia, PA, USA) and a fused-silica capillary DB-23 column (Agilent Technologies Inc.) with dimensions of 60 m × 0.25 mm (internal diameter) coated with a 0.15-µm-thick layer of 80.2% 1-methylnaphatalene. The initial oven temperature was 100 °C (hold 2 min) and was increased to 200 °C at a rate of 15 °C/min (hold 5 min). The flow rates of H_2_, air, and N_2_ make-up gas were 35, 350, and 25 mL/min, respectively. Sample volumes of 1 µL were injected at 260 °C using a split ratio of approximately 25:1. Nitrogen was used as the carrier gas at 25 mL/min. The runtime for each analysis was 12.95 min.

### 2.9. Statistical Analysis

Statistical analyses were conducted using SPSS ver. 19.0 (SPSS Inc., Chicago, IL, USA). Data were presented as mean ± SEM. Significance differences between groups in lipid accumulation, gene expression of 3T3-L1 cells and tissues, and animal experiments were determined by unpaired Student’s *t*-test. For relative abundance analysis of the gut microbiome, significant differences between groups were determined using the Wilcoxon rank-sum test. Values were considered statistically significant when *p* < 0.05.

## 3. Results

### 3.1. Anti-Adipogenic Effects of L. sakei ADM14 on Preadipocyte 3T3-L1 Cells

**Figure 1:**
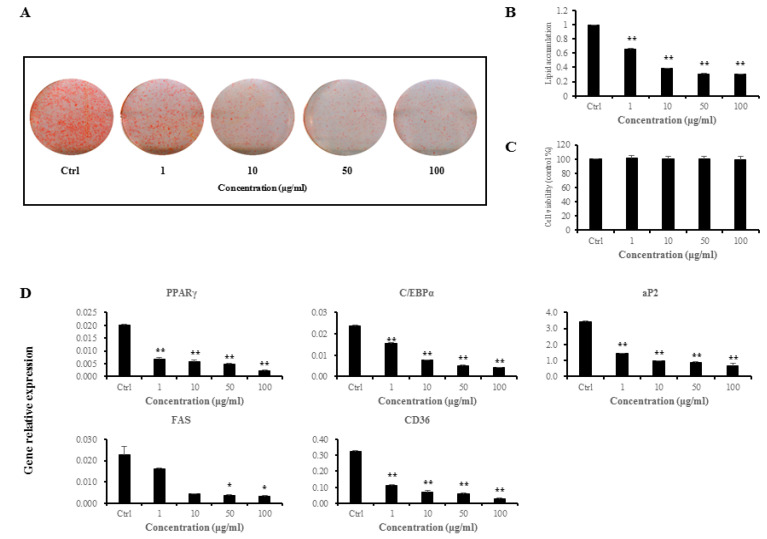
Adipogenesis inhibitory effect of *L. sakei* ADM14 in preadipocyte 3T3-L1 cells. (**A**) 3T3-L1 cells were treated with 1, 10, 50, and 100 μg/mL of *L. sakei* ADM14 extract to differentiate adipocytes for 6 days. Lipid accumulation was assessed by Oil Red O staining. (**B**) Oil Red O–stained 3T3-L1 cells were quantified. Stained cells were resolved with isopropanol, and the extracted dye was measured at 520 nm. (**C**) Effect of *L. sakei* ADM14 extract on cell viability. (**D**) *L. sakei* ADM14 suppressed the expression of adipocyte markers in 3T3-L1 cells. PPARγ, peroxisome proliferator-activated receptor-γ; C/EBPα, CCAAT-enhancer-binding protein-α; aP2, adipocyte protein 2; CD36, cluster of differentiation 36; FAS, fatty acid synthase. Data are means ± SEM. Significant differences compared with Ctrl (control), * *p* < 0.05, ** *p* < 0.01.

Pre-adipocyte 3T3-L1 cells were treated with *L. sakei* ADM14 extract at various concentrations (1, 10, 50, and 100 μg/mL) during adipocyte differentiation to measure the level of lipid accumulation. The extract was found to inhibit lipid accumulation at all concentrations treated compared to the control (Figure 1A,B). The cytotoxicity of *L. sakei* ADM14 extract against 3T3-L1 cells was tested by MTS assay, and no significant difference was observed at any concentration (Figure 1C). The expression of five genes related to adipocytes was analyzed by RT-PCR (Figure 1D) to genetically investigate whether the extract of *L. sakei* ADM14 showed concentration-dependent inhibition of adipogenesis. The expression of peroxisome proliferator-activated receptor-γ (PPARγ), CCAAT-enhancer-binding protein-α (C/EBPα), adipocyte protein 2 (aP2), and cluster of differentiation 36 (CD36) significantly decreased (*p* < 0.01) compared with the control at experimental concentrations (Figure 1D). The expression of fatty acid synthase (FAS) significantly decreased (*p* < 0.05) at concentrations of 50 and 100 μg/mL (Figure 1D).

### 3.2. Effects of L. sakei ADM14 in Indicators of Obesity in Mice

**Figure 2:**
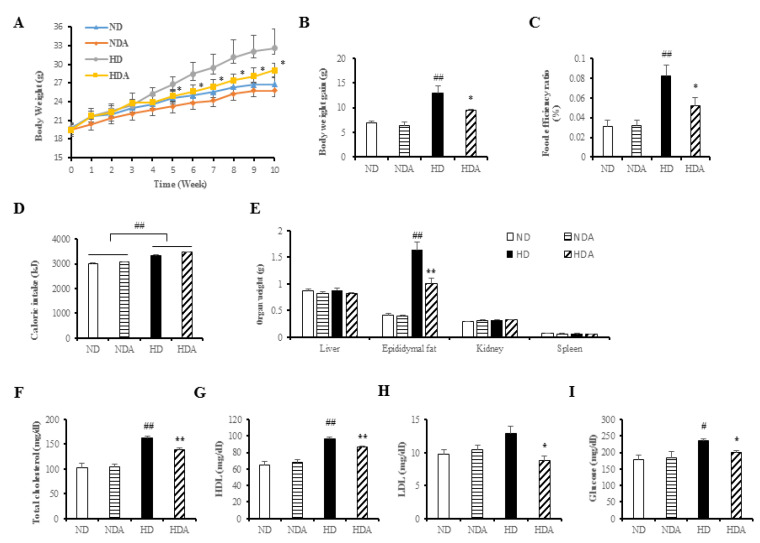
Effect of *L. sakei* ADM14 on high-fat diet-induced obese mouse model and changes in biomarkers. (**A**) Weight change of experiment mice groups for 10 weeks. (**B**) Total weight gain for each group after 10 weeks. (**C**) Food efficiency ratio over 10 weeks for each group. (**D**) Total caloric intake over 10 weeks for each group. (**E**) Organ weights of mice in each group after sacrifice. (**F**) Serum total cholesterol concentration. (**G**) Serum HDL (high density lipoprotein) concentration. (**H**) Serum LDL (low density lipoprotein) concentration. (**I**) Serum fasting glucose concentration. Results are shown as mean ± SEM (*n* = 5). Significant differences between HD and ND groups are indicated as # *p* < 0.05, ## *p* < 0.01. Significant differences between HD and HDA groups are indicated as * *p* < 0.05, ** *p* < 0.01.

Normal diets and high-fat diets were fed to each group for 10 weeks, and differences in body weights between ND and HD groups were observed (ND: 26.79 ± 0.47 g, HD: 32.57 ± 1.36 g). Significant differences in body weights were observed between HD and HDA starting at 5 weeks (Figure 2A). After 10 weeks, the HDA group (9.57 ± 0.18 g) receiving treatment with *L. sakei* ADM14 had a 25.8% lower (*p* < 0.05) body weight gain than that in the HD group (12.89 ± 1.43 g) (Figure 2B). The FER increased significantly (*p* < 0.01) in the HD compared to that in the ND but decreased by 36.7% (*p* < 0.05) in the HDA group (Figure 2C). While cumulative caloric intake was significantly different between the normal diet and high-fat diet intake groups, there was no significant difference between the HD and HDA (Figure 2D). No significant weight changes were observed in the abdominal organs including the liver and kidneys and spleen, of any group (Figure 2E). However, epididymal fat mass significantly decreased (*p* < 0.01) in the HDA compared to the HD (Figure 2E). Analysis of total blood cholesterol (Figure 2F) showed an increase of 58.8% (*p* < 0.01) in the HD compared to that in the ND. In contrast, the HDA showed a significant decrease (*p* < 0.01) compared to the HD group in total blood cholesterol. HDL (high density lipoprotein) showed a significant increase (*p* < 0.01) in HD compared to that in the ND group. In contrast, it showed a significant decrease (*p* < 0.01) of 10.68% in HDA compared to that in the HD group. LDL (low density lipoprotein) showed no significant difference between the ND and HD groups, but showed a significant decrease (*p* < 0.05) of 31.47% in HDA compared to that in the HD group. (Figure 2G,H). Analysis of fasting blood glucose concentration showed an increase of 31.4% in the HD compared to the ND (Figure 2I). The HDA showed a significant decrease (*p* < 0.05) of 15.3% in fasting blood glucose concentration compared to that in the HD group. The liver toxicity biomarkers, ALT and AST, were analyzed to identify hepatotoxicity, and there was no significant difference among the groups (Supplementary Figure S1).

### 3.3. Effects of L. sakei ADM14 in Gene Expression on Epididymal Fat Pads

**Figure 3:**
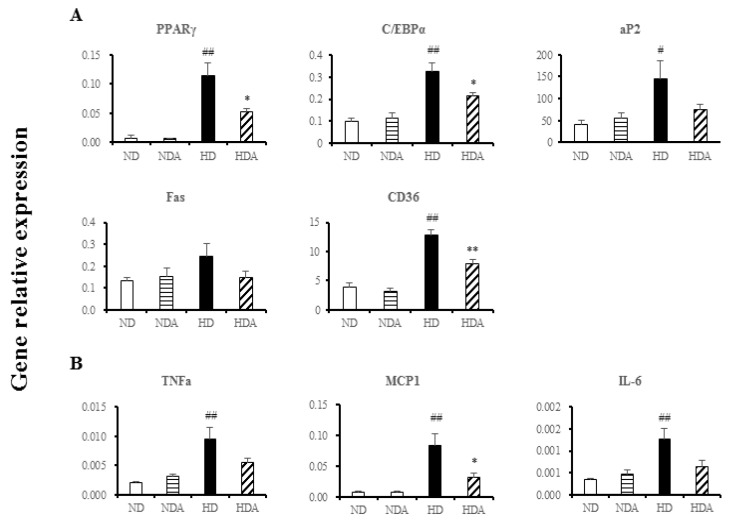
Effect of *L. sakei* ADM14 on gene expression in the epididymal fat pads. (**A**) The mRNA expression levels of PPARγ, C/EBPα, aP2, CD36, and FAS as measured by quantitative real-time polymerase chain reaction. (**B**) Effect on expression of anti-inflammatory genes measured by quantitative real-time polymerase chain reaction. PPARγ, peroxisome proliferator-activated receptor-γ; C/EBPα, CCAAT-enhancer-binding protein-α; aP2, adipocyte protein 2; CD36, cluster of differentiation 36; FAS, fatty acid synthase; TNFα, tumor necrosis factor alpha; MCP-1, monocyte chemotactic protein 1; IL-6, interleukin-6. Results are shown as mean ± SEM (*n* = 5). Significant differences between HD and ND groups are indicated as # *p* < 0.05, ## *p* < 0.01. Significant differences between HD and HDA groups are indicated as * *p* < 0.05, ** *p* < 0.01.

Expression levels of lipid-related genes in the epididymal fat pad of each group were measured by total RNA extraction followed by RT-PCR. The expression levels of four genes (except FAS) in the HD group increased significantly compared to those in the ND group (*p* < 0.01) (Figure 3A). In HDA, the gene expression of PPARγ and C/EBPα was significantly decreased compared to that of the HD group (*p* < 0.05). CD36 expression also showed a significant decrease (*p* < 0.01) (Figure 3A). However, there were no significant changes in expression of the five genes between ND and NDA groups or in the expression of aP2 and FAS genes between HD and HDA groups (Figure 3A). The expressions of pro-inflammatory genes, tumor necrosis factor alpha (TNFα), monocyte chemotactic protein 1 (MCP-1), and interleukin-6 (IL-6) were significantly increased in the HD compared to that in the ND group (*p* < 0.01) (Figure 3B). The expression of the MCP-1 gene in the HDA group decreased significantly (*p* < 0.0.5) compared with that of the HD group, and there were no significantly changes in expression of TNFα and IL-6 genes between the HDA and HD groups (Figure 3B).

### 3.4. Diversity and Composition of Cecal Microbiota

**Figure 4:**
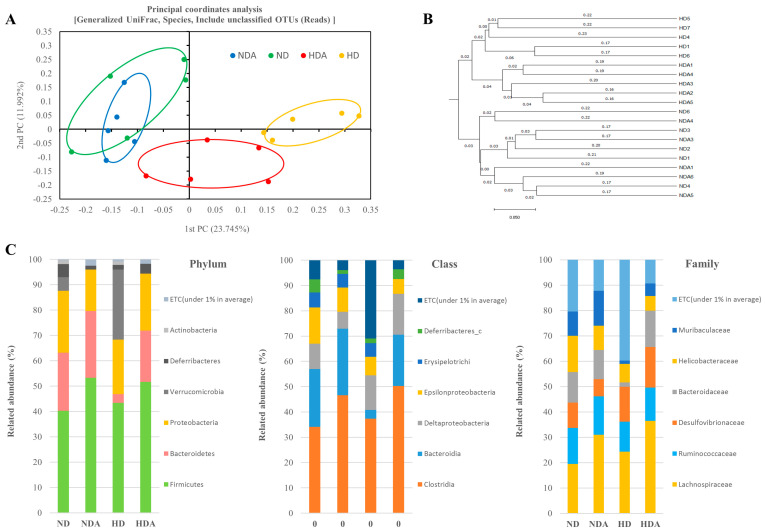
Effect of *L sakei* ADM14 on taxonomic composition. (**A**) Cecal microbiota composition of groups is shown by generalized UniFrac principal coordinates analysis (PCoA). (**B**) Unweighted Pair Group Method with Arithmetic Mean (UPGMA) clustering. The distance calculated by generalized UniFrac. (**C**) The relative abundance of the cecal microbiota at the phylum, class, and family levels. *n* = 5 per group. The nonparametric Wilcoxon signed rank test for paired data and Mann–Whitney U test for unpaired data were used.

The alpha diversity indices (Chao1 and Shannon) and beta diversity indices (PCoA and UPGMA) of the groups were calculated. No significant change in the alpha diversity index between groups was observed, and there was no significant difference in number of OTUs (Supplementary Figure S2). PCoA was performed using generalized UniFrac distances as a method to determine differences in the microbial community (Figure 4A). Distance separation between ND and NDA groups was not found, but there was clear distance separation among the normal diet groups, HD, and HDA. Differentiation between groups was clearly confirmed in cluster analysis based on UPGMA (Figure 4B). The relative abundance of bacteria in the cecum was examined to analyze how *L. sakei* ADM14 affects gut microbiota. The relative abundances of seven taxa at the phylum, class, and family levels showed distinct differences among the groups (Figure 4C). At the phyla level, the ratio of Bacteroidetes (*p* < 0.01) and Deferribacteres (*p* < 0.05) were significantly decreased in HD compared to that in the ND group. Compared with HD, the ratio of Bacteroidetes (*p* < 0.01) and Deferribacteres (*p* < 0.05) in the HDA group was significantly increased, whereas Verrucomicrobia was significantly decreased (*p* < 0.05). At the class level, the relative abundances of Bacteroidia (*p* < 0.01) and Deferribacteres_c (*p* < 0.05) were significantly reduced in the HD group compared to that in the ND. Compared to the HD group, the ratio of Bacteroidia (*p* < 0.01), Clostridia (*p* < 0.05), and Deferribacteres_c (*p* < 0.05) were significantly increased in HDA, whereas the ratio of Erysipelotrichia (*p* < 0.05) and Verrucomicrobia (*p* < 0.05) were significantly decreased. At the family level, the relative abundances of Bacteroidaceae (*p* < 0.05) and Muribaculaceae (*p* < 0.01) were significantly reduced in HD compared to that in the ND group. Compared to HD, the abundance of Bacteroidaceae (*p* < 0.01), Lachnospiraceae (*p* < 0.05), and Muribaculaceae (*p* < 0.05) showed a significant increase in the HDA group.

### 3.5. Relative Abundance of Specific Taxa in Cecum

**Figure 5:**
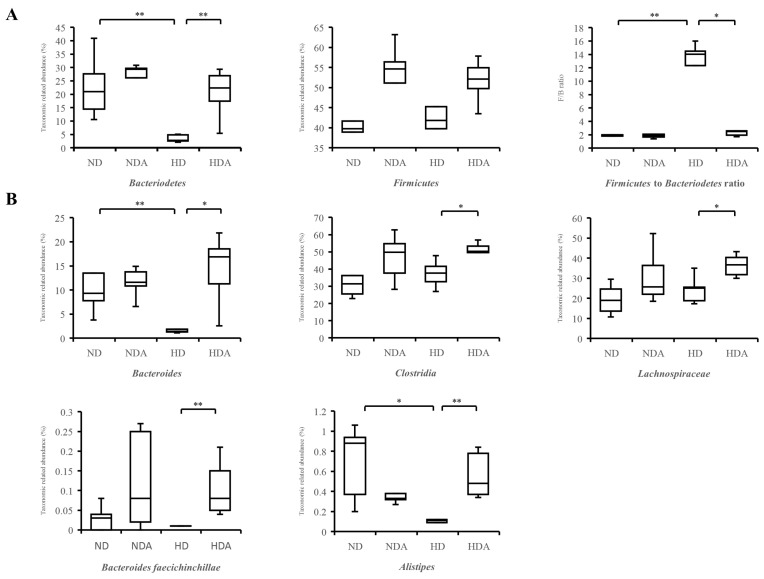
The relative abundance of specific bacteria in the cecum. (**A**) Bacteroidetes, Firmicutes, and Firmicutes to Bacteroidetes ratio. (**B**) Specific bacteria of important taxa in the human gut. The nonparametric Wilcoxon signed rank test for paired data and Mann–Whitney U test for unpaired data were used. Significant differences are indicated as * *p* < 0.05, ** *p* < 0.01.

The differences in composition of Bacteroidetes and Firmicutes at the phylum level of each group were identified (Figure 5A). Compared with ND, the relative abundance of Bacteroidetes in the HD group significantly decreased (*p* < 0.01). The relative abundance of Bacteroidetes in the HDA group, which was administered *L. sakei* ADM14, was significantly higher than that of the HD group (*p* < 0.01). No significant differences in relative abundances of Firmicutes were found between groups. The Firmicutes to Bacteroidetes ratio increased significantly (*p* < 0.01) in HD compared to that in the ND group (Figure 5A). In contrast, the Firmicutes to Bacteroidetes ratio significantly decreased (*p* < 0.05) in HDA compared with that in the HD group (Figure 5A). Changes in relative abundance of specific taxa in the cecum and known important taxa in human gut after administration of *L. sakei* ADM14 were identified (Figure 5B). Compared to the ND group, the abundance of Bacteroides in the HD group was significantly decreased (*p* < 0.01). However, the abundance of Bacteroides significantly increased (*p* < 0.05) in the HDA compared to that in the HD group. *Alistipes* also showed a similar trend, and the relative abundance decreased (*p* < 0.05) in the HD compared to that in the ND group, and increased significantly (*p* < 0.01) in HDA compared to that in the HD group. The abundance of *Bacteroides faecichinchillae* showed a significant increase (*p* < 0.01) in HDA compared to that in the HD group. Clostridia and Lachnospiraceae, which are related to the production of SCFAs, showed a significant increase (*p* < 0.05) in HDA compared to that in the HD group.

### 3.6. Concentrations of SCFAs in Cecal Contents

**Figure 6:**
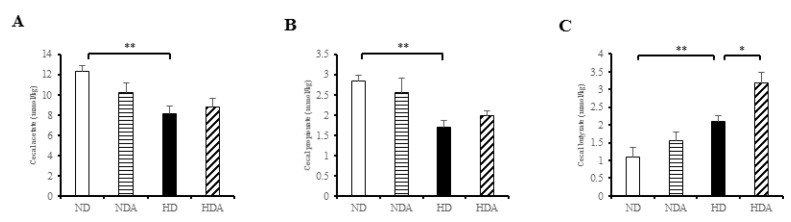
Concentrations of short-chain fatty acids (SCFAs) from cecal contents. (**A**) Acetate concentration, (**B**) propionate concentration, (**C**) butyrate concentration. SCFAs were measured by gas chromatograph. Result are shown as mean ± SEM (*n* = 4). Significant differences are indicated as * *p* < 0.05, ** *p* < 0.01.

The three short-chain fatty acids acetate, propionate, and butyrate were analyzed quantitatively in the cecum (Figure 6). Levels of acetate and propionate in the HD group were significantly lower (*p* < 0.01) than those in the ND group (Figure 6A,B), while the butyrate level in the HD group was significantly higher (*p* < 0.01) than that in the ND group (Figure 6C). At the level of acetate and propionate, there was no significant change in the HDA group. On the other hand, the level of butyrate was significantly higher compared to that in the HD group (*p* < 0.05) (Figure 6).

## 4. Discussion

Probiotic administration has been highlighted as an alternative, as pharmacological approaches to the treatment of obesity may lead to serious side effects [28,29]. Several experimental and clinical studies support the usefulness of probiotics for obesity and metabolic diseases and have demonstrated value as new strategies [29]. Probiotics have induced metabolic alleviation of obese phenotypes through host metabolism and gut microbiota modulation [30,31]. However, therapeutic approaches based on metabolites produced by probiotics or modulation of gut microbiota by probiotics must be validated by additional studies. In our previous study, we identified the anti-adipogenic effects of some lactic acid bacteria isolated from kimchi [17]. Of these, one lactic acid bacterial strain, *L. sakei* ADM14, was selected as a potential probiotic candidate based on its useful probiotic properties and good anti-adipogenic effects in in vitro assays. In our results, it was confirmed that the inhibition of adipogenesis by *L. sakei* ADM14 at specific concentrations was effective in 3T3-L1 cells. In addition, in the subsequent results, significant changes in biomarkers in high-fat diet-induced obese mice proved the utility value as probiotics.

Diet is one of the key factors generating changes in the gut microbiota profile that may contribute to obesity by affecting host energy harvesting and storage [32]. In particular, a high-fat diet has been reported to accelerate these changes leading to dysbiosis of the gut microbiota, which causes obesity and metabolic diseases [33,34]. *L. sakei* ADM14 decreased weight gain, epididymal fat mass, and levels of total blood cholesterol and fasting blood glucose in a high-fat diet group (Figure 2). For a reliable comparative analysis, there was no significant change in the results observed by administering *L. sakei* ADM14 to the normal diet group. In normal diet groups, the consistency from physiological, genetical changes and blood biomarkers showed that *L. sakei* ADM14 exerts specific effects only in situations such as high-fat and high-calorie diets. These results confirmed that *L. sakei* ADM14 has anti-obesity effects. It was shown previously that the administration of probiotics to high-fat diet-fed mice reduces fat tissue size and suppresses infiltration of pro-inflammatory macrophages into adipose tissue [35]. In addition, several studies have shown that probiotics directly affect lipid metabolism in adipose tissue by regulating the expression of lipid metabolism-related enzymes [36,37,38]. The reduction of epididymal adipose tissue mass and decrease in pro-inflammatory gene expression shown in this study agree with previous results. Just as the extract of *L. sakei* ADM14 inhibited adipogenesis in 3T3-L1 cells, the strain components such as exopolysaccharides (EPS) derived from *L. sakei* ADM14 seem to have an effect on fat tissue. It has been previously reported that components such as polysaccharides of lactic acid bacteria have several positive effects, and it is known that effects such as the downregulation of inflammation are also seen [18]. The decrease in the expression of inflammatory genes was analyzed as a positive effect of *L. sakei* ADM14 after gut colonization. Several studies have reported immuno-modulatory properties associated with anti-inflammation of *L. sakei* [14,15,39]. As a result, *L. sakei* ADM14 was expected to have anti-obesity effects by inhibiting lipid accumulation in adipose tissue while alleviating chronic hypo-inflammation in adipose tissue (Figure 2 and Figure 3). Increased levels of total blood cholesterol and blood glucose are generally associated with obesity and dyslipidemia, which is consistently observed in the diet-induced obesity model [40]. Several probiotic strains have been reported to lower blood cholesterol levels [41]. *L. sakei* ADM14 has been shown to reduce the risk of obesity-related factors while lowering total cholesterol, LDL, and fasting blood glucose levels (Figure 2). It has been reported that the remaining cholesterol not absorbed by the small intestine is converted into other metabolites by the gut microbiota, which helps eliminate cholesterol in the body [42]. In this study, the gut microbiota modulated by *L. sakei* ADM14 appears to lower blood cholesterol levels. Overall, our results demonstrated that treatment with *L. sakei* ADM14 shows several beneficial effects in an obese mouse model induced by a high-fat diet.

Several studies have confirmed changes in the diversity and composition of gut microbiota in obesity states, but the criteria and profile of the gut microbiota of healthy or unhealthy hosts have not been established [43,44]. In this study, no significant differences in diversity indices and richness estimation of the cecal microbiota were found between groups, but there were clear differences in the composition of the dominant microbial community in the cecum between ND and HD groups and between HD and HDA groups (Figure 4). Constitutive dysbiosis of gut microbiota after long-term administration of a high-fat diet was observed compared to the normal diet group (Figure 4). The change was particularly noticeable in the proportion of dominant bacterial groups such as Firmicutes and Bacteroidetes. In addition, an imbalance in energy harvesting can occur due to changes in the Firmicutes to Bacteroidetes ratio [45]. The relative abundance ratio of Firmicutes to Bacteroidetes from various studies seems to be an index of obesity sensitivity [46] as shown in the results of the present study. The significant increase in the Firmicutes to Bacteroidetes ratio of the HD group is estimated to cause increased obesity sensitivity and imbalance in energy harvesting. In the HDA group administered *L. sakei* ADM14, the proportion of Bacteroidetes increased, and thereby the Firmicutes to Bacteroidetes ratio decreased compared to that in the HD group, and the imbalance in energy harvest seems to have improved (Figure 4 and Figure 5). This change in the dominant strains Firmicutes and Bacteroidetes is one of the key factors of the change in biomarkers in our experimental mouse model. It is known from reported studies [6,33] that the obese microbiome has an increased ability to harvest energy from their diet compared to normal microbiome. The increased energy is directed and accumulated in the liver and body fat tissues. In our results, it was confirmed that the ratio of Firmicutes to Bacteroidetes in the HD group was excessively high compared to that in the ND group, and an extreme change occurred in terms of energy harvesting. The significant increase in body fat weight supports this. On the other hand, the data shown in the HDA group showed that the significant change in the ratio of Firmicutes to Bacteroidetes lowered by administration of *L. sakei* ADM14 was reversed, unlike the situation of the HD group. At more detailed taxonomic levels, several bacterial taxa have been reported to be associated with dysbiosis in obesity, and our results also showed changes in several bacterial taxa [47] (Figure 5). *Bacteroides faecichinchillae* are reported to be one of the abundant bacteria in stool samples of non-obese people compared to those of obese people and to have a strong relationship with lean body type (BMI less than 20) [48]. Additionally, *Alistipes* present in fecal samples showed a negative correlation with various clinical indicators including body weight and biochemical indices in serum lipids [49]. From the results in this study, we posit that the relative abundance of *Bacteroides faecichinchillae* and *Alistipes* in the cecum of the HDA group affects the alleviation of obesity. Among them, the abundance of *Alistipes* was reported to be a strain that is closely related to health and is affected by the gut dysbiosis of metabolic diseases such as obesity and non-alcoholic fatty hepatitis [50]. In our results, the re-increase of *Alistipes* in the HDA group, which was decreased in the HD group, may be considered as a clue to the recovery of this gut dysbiosis. These changes in specific gut microbiota and taxa of known importance in the human gut correlate with physical biomarker results in the obese mouse model, confirming that obesity inhibition is exerted.

Some taxa of gut microbiota can digest carbohydrates such as low-digestible polysaccharides and dietary fiber to produce short-chain fatty acids (SCFAs) such as acetate, propionate, and butyrate [51,52]. *Lactobacillus* species are not a direct producer of SCFAs, but they can indirectly improve the productivity of SCFAs through gut microbiota modulation [53]. SCFAs serve as an energy source, act as signaling molecules, and play a beneficial role in host health as well as immune support [54,55]. In the rodent obese model, ingestion of butyrate and acetate has been reported to suppress animal weight gain [56,57]. In addition, studies have shown that administration of butyrate and propionate suppresses food intake and weight gain due to high-fat diet, and prevents glucose intolerance [56,58,59]. In particular, the beneficial effects of butyrate on intestinal homeostasis and energy metabolism have been noted [60]. Our results showed that the change in microbial taxa related to SCFA production might occur due to gut microbiota modulation by *L. sakei* ADM14. The proportions of Clostridia and Lachnospiraceae, the central producers of intestinal butyrate [61,62], showed significant increases in the HD group administered *L. sakei* ADM14, suggesting that *L. sakei* ADM14 influenced the expansion of both taxa. There were significant changes in the relative abundance of *Bacteroides* between HD and HDA groups in this study. *Bacteroides* species can degrade a wide range of complex carbohydrates that provide a carbon source to SCFAs [51]. At the same time, *Bacteroides* species are the main producers of acetate and propionate [63]. In our results, a low relative abundance of *Bacteroides* was found in the high-fat diet group in which the concentrations of acetate and propionate were significantly lower than those of the normal diet group (Figure 5). The groups administered *L. sakei* ADM14 showed significant increases in butyrate concentration and a correlation with the relative abundance of Clostridia and Lachnospiraceae. The concentration of these SCFAs and the linkage of specific taxa prove that *L. sakei* ADM14 can modulate the gut microbiota and at the same time improve the butyrate production capacity. Like the above-mentioned efficacy of SCFAs, the elevated concentrations of butyrate are believed to have contributed to the suppression of weight gain in the obese mouse model. Collectively, *L. sakei* ADM14 affected obesity by lowering the risk of metabolic problems through changes in biomarkers of a diet-induced obese mouse model and strongly modulated the cecal microbiota. However, additional studies are needed to demonstrate the effect on metabolic problems other than obesity and the changes in microbial composition in the overall intestinal tract including the large intestine through the administration of *L. sakei* ADM14.

## 5. Conclusions

This study showed that metabolic and microbiome changes caused during a high-fat diet in a mouse model can induce obesity. We confirmed attenuation of the obesity-related biomarkers, such as significant decrease in adipose tissue and reduction of total cholesterol and fasting blood glucose, in an experimental model with administration of *L. sakei* ADM14. Changes in these factors showed a strong correlation with *L. sakei* ADM14 gut microbiota modulation along with positive changes such as increased concentration of butyrate in the cecum. Therefore, the potential probiotic *L. sakei* ADM14 brings health benefits to the host by modulating gut microbiota and ability to stimulate SCFA production. It may be useful as an alternative supplement to alleviate metabolic syndrome and obesity caused by dysbiosis. At the same time, it was proved that there were no significant changes and adverse effects in the normal diet group, and the stability of *L. sakei* ADM14 was confirmed. However, further research is needed to determine the impact on the overall gastrointestinal tract, including the large intestine, and gut environmental changes. In addition, there is a need to increase the value of probiotics’ utility by evaluating their effects on other metabolic diseases.

## Supplementary Materials

The following are available online at https://www.mdpi.com/2072-6643/12/12/3703/s1, Figure S1: Effect of *L. sakei* ADM14 on the toxicity biomarkers. (A) Serum ALT. (B) Serum AST. Results are shown as mean ± SEM (*n* = 5). Figure S2: Alpha diversity indexes between groups. (A) The Chao1 richness estimator. (B) Shannon’s diversity index. (C) The number of operational taxonomic units (OTUs). The nonparametric Wilcoxon signed rank test for paired data. Results are shown as mean ± SEM (*n* = 5). Table S1: Specific primer sequence. PPARγ, peroxisome proliferator-activated receptor-γ; C/EBPα, CCAAT-enhancer-binding protein-α; aP2, adipocyte protein 2; CD36, cluster of differentiation 36; FAS, fatty acid synthase; TNFα, tumor necrosis factor alpha; MCP-1, monocyte chemotactic protein 1; IL-6, interleukin-6; 36B4, acidic ribosomal phosphoprotein P0.
