# Supplementary material for: Lactobacillus sakei ADM14 Induces Anti-Obesity Effects and Changes in Gut Microbiome in High-Fat Diet-Induced Obese Mice
Source: Nutrients. 2020 Nov 30;12(12):3703. doi: 10.3390/nu12123703 (PMC7761388; doi:10.3390/nu12123703)
Supplement: Supplementary file 1 [file nutrients-12-03703-s001.pdf]

Supplementary Materials

Lactobacillus sakei ADM14 Induces Anti-Obesity Effects and Changes in Gut Microbiome in High-fat Diet-induced Obese Mice

Sung-Min Won, Siyu Chen, Seo Yeon Lee, Kyung Eun Lee, Kye Won Park, Jung-Hoon Yoon

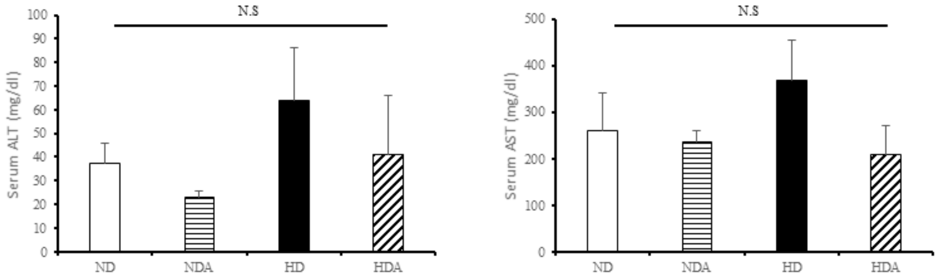

Figure S1. Effect of *L sakei* ADM14 on the toxicity biomarkers. (A) Serum ALT. (B) Serum AST. Results are shown as mean ± SEM (n = 5).

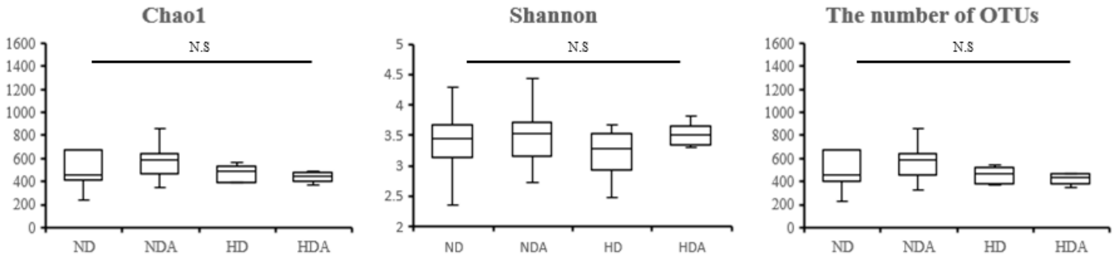

Figure S2. Alpha Diversity Indexes Between Groups. (A) The Chao1 richness estimator. (B) Shannon's diversity index. (C) The number of OTUs. The nonparametric Wilcoxon signed rank test for paired data. Results are shown as mean ± SEM (n = 5).

Supplementary table1. Specific primer sequence. PPARγ, peroxisome proliferator-activated receptor-γ; C/EBPα, CCAAT-enhancer-binding protein-α; aP2, adipocyte protein 2; CD36, cluster of differentiation 36; FAS, fatty acid synthase; TNFα, tumor necrosis factor alpha; MCP-1, monocyte chemotactic protein 1; IL-6, interleukin-6; 36B4, acidic ribosomal phosphoprotein P0.

**Gene (mouse)**	**Primer Sequence**
PPARγ	F: 5′- CCAGAGCATGGTGCCTTCGC -3′<br>R: 5′- CAGCAACCATTGGGTCAGCTC -3′
C/EBPα	F: 5′- GAACAGCAACGAGTACCGGGTA -3′<br>R: 5′- GCCATGGCCTTGACCAAGGAG -3′
FAS	F: 5′- GCTGCTGTTGGAAGTCAGC -3′<br>R: 5′- AGTGTTCGTTCCTCGGAGTG -3′
aP2	F: 5′- CACCGCAGACGACAGGAAG -3′<br>R: 5′- GCACCTGCACCAGGGC -3′
CD36	F: 5′- GGCCAAGCTATTGCGACAT -3′<br>R: 5′- CAGATCCGAACACAGCGTAGA -3′
TNFα	F: 5′- GCCACCACGCTCTTCTGCCT -3′<br>R: 5′- GGCTGATGGTGTGGGTGAGG -3′
MCP-1	F: 5′- TCTGGACCCATTCCTTCTTG -3′<br>R: 5′- AGGTCCCTGTCATGCTTCTG -3′
IL-6	F: 5′- CAAGAAAGACAAAGCCAGAGTCCTT -3′<br>R: 5′- TGGATGGTCTTGGTCCTTAGCC -3′
36B4	F: 5′- AGATGCAGCAGATCCGCAT-3′<br>R: 5′- GTTCTTGCCCATCAGCACC-3′
